# β-oxidation-mediated differentiation of effector CD4^+^ T cells in the lung enhances neuroinflammation

**DOI:** 10.1186/s12974-026-03865-5

**Published:** 2026-05-09

**Authors:** Qianling Jiang, Gaochen Zhu, Xin Ma, Wen Si, Guan Yang

**Affiliations:** 1 Department of Infectious Diseases and Public Health, Jockey Club College of Veterinary Medicine and Life Sciences, City University of Hong Kong, Kowloon, 999077 Hong Kong SAR China; 2 Shenzhen Research Institute, City University of Hong Kong, Shenzhen, 518057 China

**Keywords:** Multiple sclerosis, Experimental autoimmune encephalomyelitis, Lung-brain axis, Lipid metabolism, Effector CD4^+^ T cells

## Abstract

Although the lung-brain axis has emerged as a potential regulator of central nervous system (CNS) autoimmunity, the cellular and molecular mechanisms by which the lung microenvironment influences pathogenesis of multiple sclerosis (MS) remain unclear. Here, using experimental autoimmune encephalomyelitis (EAE), a murine model of MS, we found a marked expansion of effector CD4⁺ T cells in the lungs of EAE mice. The EAE lung microenvironment promoted metabolic reprogramming in CD4⁺ T cells, characterized by enhanced fatty acid uptake and upregulation of carnitine transporters. Metabolomic profiling further demonstrated enrichment of carnitine-related metabolites in the EAE lungs, with a strong correlation between metabolic profiles in the lungs and brains, suggesting coordinated metabolic remodeling along the lung-brain axis. Mechanistically, the EAE lung microenvironment significantly enhanced effector CD4⁺ T cell differentiation in vitro through a β-oxidation-dependent pathway. Importantly, pharmacological inhibition of β-oxidation in the lungs significantly attenuated EAE severity, reduced CD4⁺ T cell infiltration into the CNS, and impaired effector CD4⁺ T cell differentiation in the lungs. Collectively, these findings demonstrate that β-oxidation-mediated differentiation of effector CD4⁺ T cells in the lung exacerbates neuroinflammation, highlighting the lung-brain axis as a potential therapeutic target for MS.

## Introduction

The autoimmune processes occurring within the central nervous system (CNS) are influenced not only by local neuroinflammatory conditions but also by peripheral organs. Emerging clinical and epidemiological studies have highlighted the underappreciated role of the lung-brain axis-the bidirectional communication and interaction between the lung and the brain-in the pathogenesis of multiple sclerosis (MS), a chronic autoimmune disease of the CNS [1]. In particular, smoking and respiratory infections caused by influenza and herpesviruses are associated with an increased risk of MS and exacerbation of the disease [2–4]. Utilizing the experimental autoimmune encephalomyelitis (EAE) model of MS, researchers have further elucidated the significance of the lung-brain axis in CNS autoimmunity. For instance, influenza infection of the upper respiratory tract can induce spontaneous EAE symptoms in 2D2 TCR transgenic mice, implicating respiratory pathogens in the initiation of neuroinflammatory cascades [5]. Additionally, intranasal administration of lipopolysaccharide could delay the onset of EAE [6] and modulating lung immune cells via pulmonary delivery of antigen-specific nanoparticles could ameliorate EAE symptoms [7]. These findings together underscore a complex interplay between the lung and brain in MS/EAE, suggesting that pulmonary factors may drive neuroinflammation and disease progression. However, the role of the lung-brain axis in MS/EAE remains poorly understood, and its therapeutic potential warrants further investigation.

The important role of CD4⁺ T cells are well-established in EAE [8]. Following activation by antigens in the periphery, these cells migrate across the blood-brain barrier and stimulate resident microglia and infiltrating macrophages. Together, these immune cells contribute to oligodendrocyte loss and myelin damage [8]. This understanding has prompted a focus on identifying the peripheral sites and signals that equip CD4⁺ T cells with pathogenic properties prior to enter the CNS. Notably, a previous study has indicated that encephalitogenic T cells can be licensed in the lung, where they undergo transcriptional reprogramming and acquire migratory capabilities toward the CNS [9]. Despite these insights, the phenotypic and functional characteristics of CD4⁺ T cells in the lung during EAE, as well as the mechanisms through which the lung microenvironment influences their functional shift, remain largely unexplored.

In this study, we observed a marked expansion of effector CD4⁺ T cells in the lungs of EAE mice and further characterized CD4⁺ T cell subclusters using single-cell RNA sequencing. CD4⁺ T cells from the lungs of EAE mice exhibited enhanced lipid uptake and upregulation of carnitine transporters, coinciding with altered lipid metabolism in the lung microenvironment. Besides, we found that bronchoalveolar lavage fluid (BALF) from EAE mice promoted the differentiation of effector CD4⁺ T cells, an effect that was abolished by the inhibition of β-oxidation. Moreover, blocking β-oxidation in the lung by etomoxir ameliorated the severity of EAE and impaired effector CD4⁺ T cell differentiation. Interestingly, the lung metabolome exhibited a high degree of similarity to the brain metabolic profile in EAE mice. Together, these results demonstrate that β-oxidation-mediated differentiation of effector CD4⁺ T cells in the lung exacerbates neuroinflammation, underscoring the lung-brain axis as a promising therapeutic target for MS/EAE.

## Results

### Effector CD4^+^ T cells accumulate in the lungs of EAE mice

**Fig. 1:**
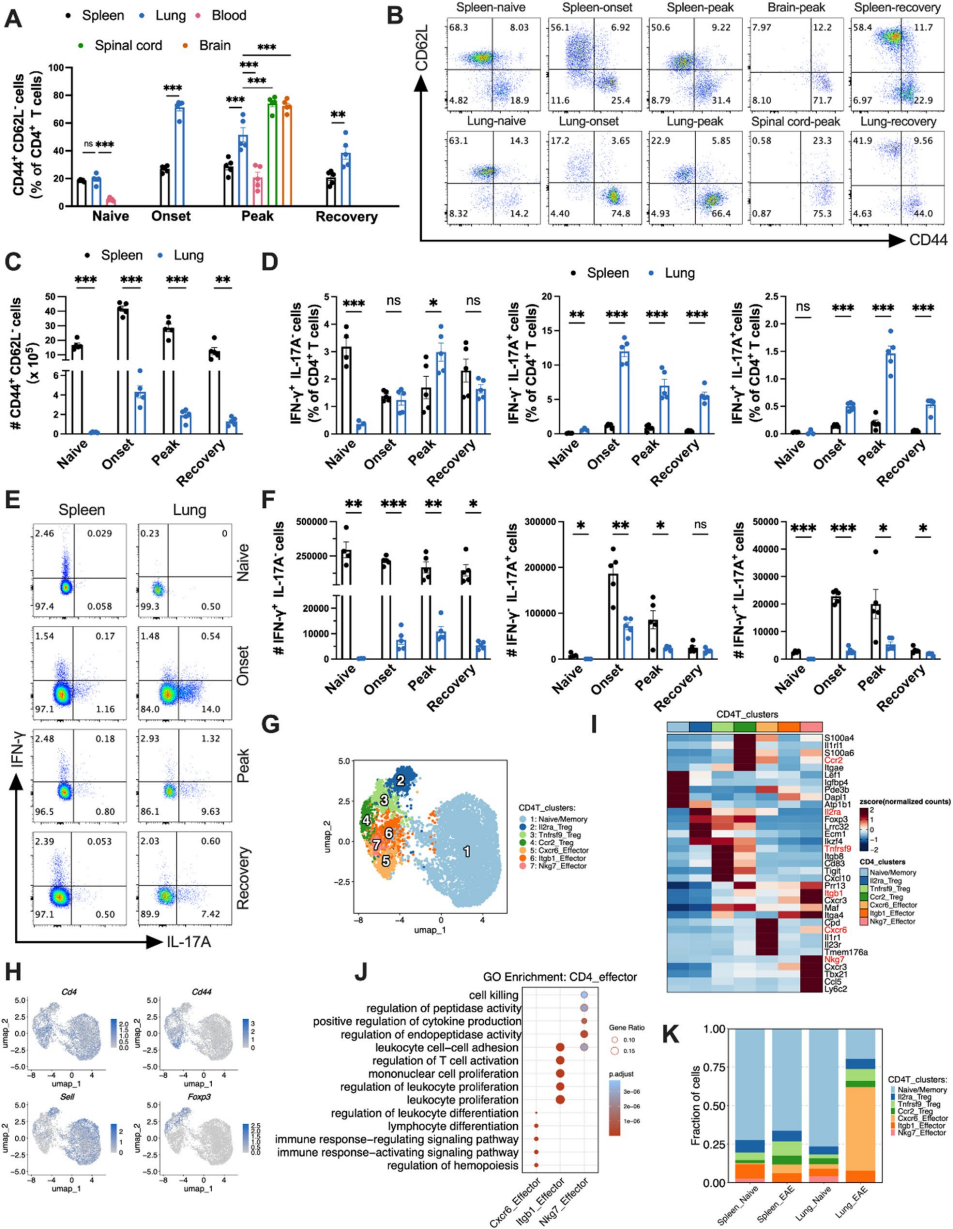
Accumulation of effector CD4^+^ T cells in the lungs of EAE mice.** A-C** Frequencies (**A**), representative flow cytometry plots (**B**), and absolute numbers (**C**) of effector CD4⁺ T cells (CD44⁺ CD62L⁻) in various tissues from naïve (untreated) mice and EAE mice at disease onset (10 dpi), peak (19 dpi), and recovery (35 dpi) (*n* = 5). **D-F **Frequencies (**D**), representative flow cytometry plots (**E**), and absolute numbers (**F**) of IFN-γ-producing, IL-17A-producing, and IFN-γ- and IL-17A- dual-producing CD4⁺ T cells in various tissues from naïve (untreated) mice and EAE mice at disease onset (10 dpi), peak (19 dpi), and recovery (35 dpi) (*n* = 3–5). **G** UMAP of CD4^+^ T cell subclusters in the spleens and lungs of naïve and EAE mice (19 dpi). **H **Expression of selected marker genes in CD4⁺ T cells from the spleens and lungs of naïve and EAE mice, including *Cd4*, *Cd44*, *Sell*, and *Foxp3*. **I **Top five marker genes identified in CD4⁺ T cell subclusters. **J** Gene Ontology (GO) enrichment analysis of effector CD4⁺ T cell subclusters. **K** Frequencies of CD4⁺ T cell subclusters from single-cell RNA sequencing analysis. Data are mean ± SEM. Statistical analyses were performed using one-way ANOVA with Tukey’s multiple comparisons test (**A**-naïve and peak stages), and unpaired Student’s t-test (**A**-onset and recovery stages, **C**, **D** and **F**). *P < 0.05, **P < 0.01, and ***P < 0.001

To better characterize CD4⁺ T cells in the lungs of EAE mice, we first assessed their functional status and compared them with those from the spleens across different disease stages, including naïve (untreated), disease onset (10 dpi), peak (19 dpi), and recovery (35 dpi). We found that the frequency of effector CD4⁺ T cells (CD44⁺ CD62L⁻) was markedly increased in the lungs during EAE, reaching the highest level at disease onset, followed by a modest decrease at the peak and recovery stages (Fig. 1A, B). In contrast, only a slight increase in effector CD4⁺ T cells was observed in the spleens during EAE (Fig. 1A, B). Importantly, while the frequency of effector CD4⁺ T cells was comparable between lungs and spleens under naïve conditions, the lung consistently exhibited a higher proportion of effector CD4⁺ T cells at all EAE stages examined (Fig. 1A, B). Although the absolute number of effector CD4⁺ T cells increased in both organs during EAE, the spleens consistently showed higher absolute numbers than the lungs, due to its substantially larger total CD4⁺ T cell pool (Fig. 1C). Therefore, these data indicate a more pronounced enrichment and dynamic change of effector CD4⁺ T cells in the pulmonary compartment compared with the spleen during disease progression. Furthermore, CD4⁺ T cells in the brain and spinal cord were predominantly of the effector phenotype (Fig. 1A, B), underscoring the central role of effector CD4⁺ T cells in EAE and suggesting that their differentiation state may be shaped in a tissue-specific manner across peripheral organs and the CNS.

Given the established pathogenic roles of Th1 and Th17 cells in EAE [8], we next sought to determine whether CD4⁺ T cells in the lungs of EAE mice exhibit altered Th1 or Th17 polarization compared with those from naïve mice. We found that the frequencies of IFN-γ-producing, IL-17A-producing, and IFN-γ- and IL-17A- dual-producing CD4⁺ T cells were all increased in the lungs during EAE, with distinct temporal kinetics among subsets (Fig. 1D, E). In contrast, these populations showed only minor changes in the spleens (Fig. 1D, E). Notably, IL-17 A-producing CD4⁺ T cells were markedly enriched in the lungs compared with the spleens during EAE (Fig. 1D, E). Consistently, the absolute numbers of IFN-γ-producing, IL-17A-producing, and IFN-γ- and IL-17A- dual-producing CD4⁺ T cells were increased in the lungs relative to naïve controls (Fig. 1F). Although the spleens contained higher absolute numbers of these subsets due to its larger total CD4⁺ T cell pool, the lung exhibited a stronger relative enrichment (Fig. 1F). Overall, these results indicate that the lung represents a site of preferential expansion and polarization of pathogenic Th1/Th17 responses during EAE.

As CD4⁺ T cells are a heterogeneous population, we next performed single-cell RNA sequencing to further characterize CD4⁺ T cells from the lungs of naïve and EAE mice (19 dpi), and compared them with those from the spleen. CD4⁺ T cells were unbiasedly clustered into seven subpopulations and annotated based on marker gene expression: naïve/memory, *Il2ra*_Treg, *Tnfrsf9*_Treg, *Ccr2*_Treg, *Cxcr6*_effector, *Itgb1*_effector, and *Nkg7*_effector (Fig. 1G-I). Gene Ontology (GO) enrichment analysis further revealed functional specialization among these effector subsets, with *Cxcr6*_effector CD4⁺ T cells associated with pathways related to immune activation and inflammatory response regulation, *Itgb1*_effector CD4⁺ T cells enriched in cell proliferation programs, and *Nkg7*_effector CD4⁺ T cells enriched in cell-killing pathways (Fig. 1J). Notably, *Cxcr6*_effector CD4⁺ T cells were markedly expanded in the lungs of EAE mice, whereas the proportion of *Nkg7*_effector cells was reduced during disease (Fig. 1K). Together, these results indicate that EAE is associated with a selective reshaping of the CD4⁺ T cells in the lungs, characterized by the preferential expansion of the inflammatory *Cxcr6*⁺ effector subset.

### Lung microenvironment in EAE mice drives lipid metabolic reprogramming in CD4⁺ T cells

**Fig. 2:**
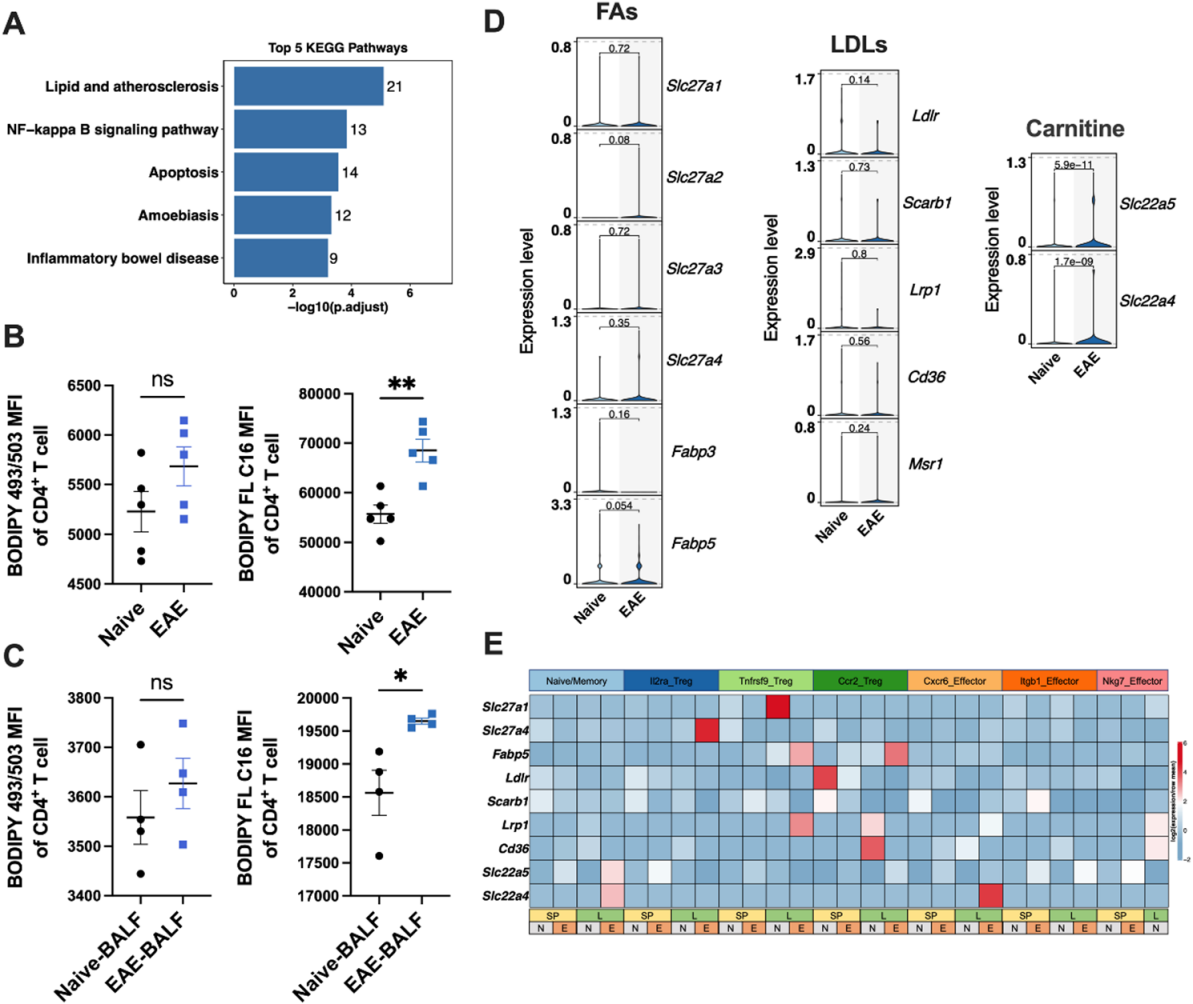
CD4⁺ T cells in the lungs of EAE mice show alterations in lipid metabolism. **A** KEGG pathway enrichment analysis of upregulated differentially expressed genes in CD4⁺ T cells from the lungs of EAE mice compared with naïve mice. **B** MFI of BODIPY 493/503 staining and BODIPY FL C16 uptake in CD4⁺ T cells from the lungs of naïve and EAE mice (19 dpi) (*n* = 5). **C** MFI of BODIPY 493/503 staining and BODIPY FL C16 uptake in anti-CD3e/CD28-stimulated naïve CD4⁺ T cells cultured in vitro with BALF collected from naïve or EAE mice (19 dpi) (*n* = 4). **D** Expression levels of lipid uptake-associated genes in CD4⁺ T cells from the lungs of naïve and EAE mice. **E** Expression levels of lipid uptake-associated genes in CD4⁺ T cell subclusters from the spleens and lungs of naïve and EAE mice (19 dpi). Data are mean ± SEM. Statistical analyses were performed using unpaired Student’s t-test (**B**-**C**), and Wilcoxon rank-sum test (**D**). *P < 0.05 and **P < 0.01

To further investigate global functional alterations in CD4⁺ T cells following EAE induction, we conducted KEGG enrichment analysis on genes upregulated in CD4⁺ T cells from the lungs of EAE mice compared with their naïve counterparts. Our analysis revealed that CD4⁺ T cells from EAE mice were mainly enriched in lipid and atherosclerosis pathway and NF-κB signaling pathway (Fig. 2A), suggesting that the lung microenvironment in EAE promotes both metabolic and inflammatory reprogramming in these cells. Based on this finding, we next assessed lipid accumulation and fatty acid uptake in lung CD4⁺ T cells. We found that the total neutral lipid content, measured by BODIPY 493/503 (a neutral lipid dye), showed only a modest and non-significant increase in CD4⁺ T cells from the lungs of EAE mice (19 dpi) (Fig. 2B). In contrast, these cells exhibited significantly higher fatty acid uptake, as assessed by BODIPY FL C16 (a fluorescent long-chain fatty acid analog), compared with naïve controls (Fig. 2B). To directly test whether the lung milieu in EAE mice promotes lipid metabolic reprogramming in CD4⁺ T cells, we cultured naïve CD4⁺ T cells (CD44⁻ CD62L⁺) with BALF collected from either naïve or EAE mice (19 dpi). Naïve CD4⁺ T cells exposed to BALF from EAE mice displayed increased fatty acid uptake while maintaining comparable levels of total neutral lipids compared with those treated with BALF from naïve mice (Fig. 2C).

Lipid-related metabolites enter cells through several mechanisms. Free fatty acids are imported via membrane transporters, supporting their incorporation into metabolic pathways and cellular functions [10]. Lipoprotein-associated lipids, such as low-density lipoprotein and oxidized low-density lipoprotein, are internalized through scavenger receptors and processed to provide cholesterol and fatty acids for intracellular pools [11]. Additionally, carnitine uptake through high‑affinity transporters enables acylcarnitine formation, which shuttles fatty acids from the cytosol into the mitochondrial matrix for β-oxidation, linking lipid uptake to mitochondrial energy production [12]. To determine which of these pathways is engaged in the function of lung CD4⁺ T cells during EAE, we examined the expression of associated genes. We observed a significant upregulation of genes with carnitine uptake (*Slc22a5* and *Slc22a4*), but not those involved in the uptake of fatty acids (*Slc27a1-4*, *Fabp3*, and *Fabp5*) or low-density lipoprotein (*Ldlr*, *Scarb1*, *Lrp1*, *Cd36*, and *Msr1*), in CD4⁺ T cells from the lungs of EAE mice (Fig. 2D). To determine whether these metabolic changes are specific to lung CD4⁺ T cells, we compared the expression of these genes across CD4⁺ T cell subsets from the lungs and spleens under both naïve and EAE conditions. Remarkably, we found that the carnitine transporter gene *Slc22a4* was highly enriched in *Cxcr6*⁺ effector CD4⁺ T cells from the lungs of EAE mice (Fig. 2E). In addition, both *Slc22a4* and *Slc22a5* were upregulated in naïve/memory CD4⁺ T cells from the lungs of EAE mice compared with those from naïve lungs, as well as from the spleens under both naïve and EAE conditions (Fig. 2E). Together, these results indicate that the lung microenvironment in EAE mice promotes metabolic reprogramming in CD4⁺ T cells, characterized by enhanced fatty acid uptake and upregulation of carnitine transporters. This effect is preferentially observed in EAE lung CD4⁺ T cell subsets, particularly within *Cxcr6*⁺ effector cells, supporting a lung-specific metabolic adaptation in EAE.

### Lung microenvironment in EAE mice promotes differentiation of effector CD4⁺ T cells

**Fig. 3:**
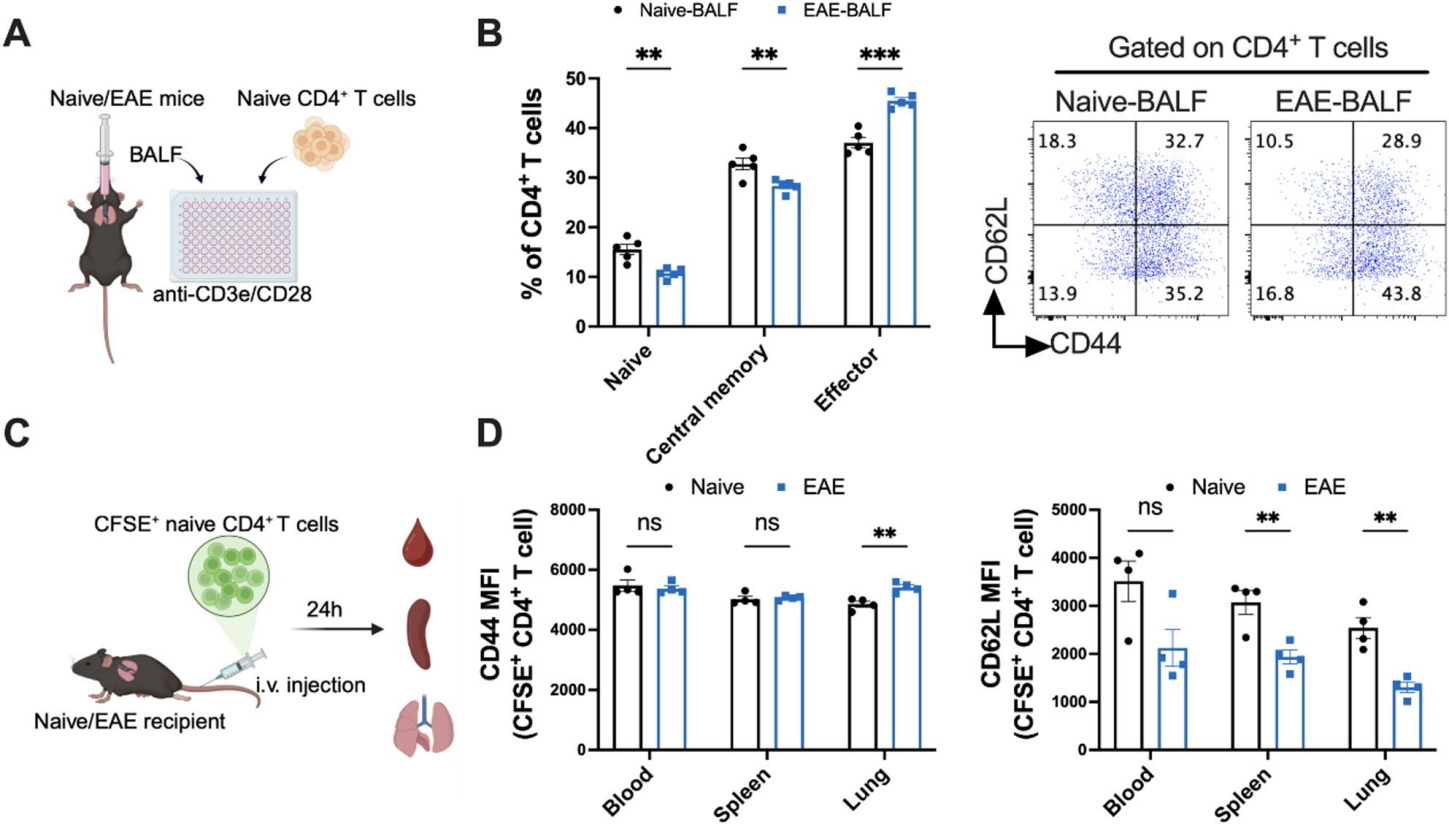
The lung microenvironment promotes effector CD4⁺ T cell differentiation in EAE mice.** A** Schematic illustration of the in vitro differentiation of naïve CD4⁺ T cells with BALF from naïve or EAE mice (19 dpi) under anti-CD3e/CD28 stimulation for 24 h. **B** Frequencies and representative flow cytometry plots of effector CD4⁺ T cells generated under the conditions shown in (**A**) (*n* = 5). **C** Schematic illustration of the in vivo transfer of CFSE-labeled naïve CD4⁺ T cells into naïve or EAE mice (19 dpi). Blood, spleens, and lungs were collected 24 h later to assess the differentiation status of CFSE⁺ CD4⁺ T cells. **D **MFI of CD44 and CD62L on CFSE⁺ CD4⁺ T cells isolated from the blood, spleens, and lungs of recipient mice (*n* = 4). Data are mean ± SEM. Statistical analyses were performed using unpaired Student’s t-test (**B** and **D**). **P < 0.01, and ***P < 0.001

Tissue-specific environments significantly influence T cell responses [13–15]. To assess whether the lung microenvironment in EAE mice promotes effector CD4⁺ T cell differentiation, naïve CD4⁺ T cells were stimulated with anti-CD3e/CD28 and cultured with BALF collected from naïve or EAE mice (19 dpi) for 24 h (Fig. 3A). Notably, BALF from EAE mice markedly increased the proportion of effector CD4⁺ T cells compared to that from naïve mice (Fig. 3B). To further evaluate the impact of the lung microenvironment on CD4^+^ T cell responses in vivo, we labeled naïve CD4⁺ T cells with carboxyfluorescein succinimidyl ester (CFSE) and transferred them intravenously into naïve or EAE mice (19 dpi). Blood, spleens, and lungs were collected 24 h later to assess the differentiation status of CFSE⁺ CD4⁺ T cells (Fig. 3C). We found that CFSE⁺ CD4⁺ T cells isolated from the lungs of EAE mice exhibit increased CD44 expression compared to those from naïve mice, whereas no such difference was observed in the blood or spleens (Fig. 3D). In addition, CFSE⁺ CD4⁺ T cells from all three tissues showed decreased CD62L expression in EAE mice (Fig. 3D). These findings indicate that the lung microenvironment in EAE mice more effectively induces naïve CD4⁺ T cells into an effector state compared to the spleen or blood.

### EAE induction alters the lung metabolome with perturbations in lipid metabolism

**Fig. 4:**
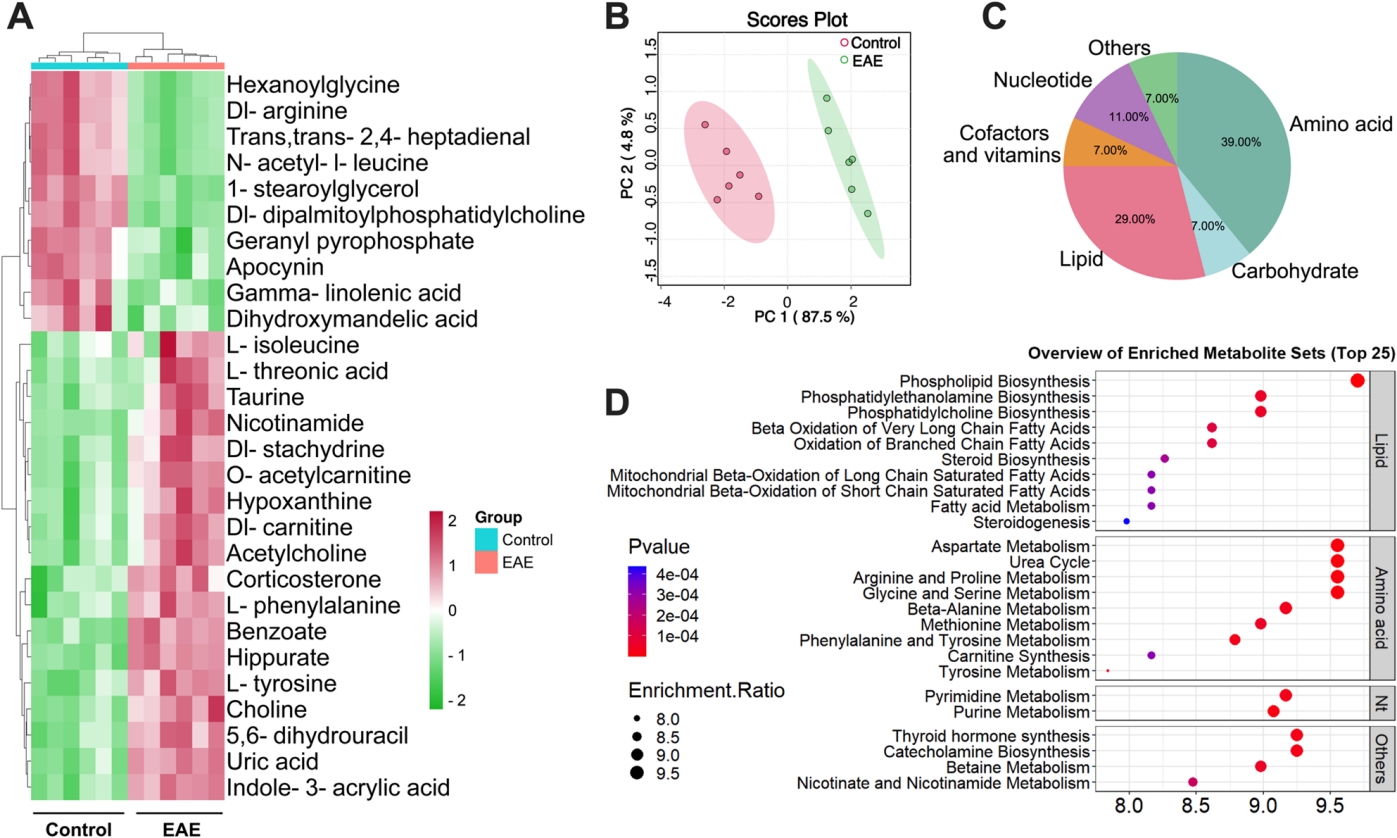
Altered metabolic profile in the BALF of EAE mice.** A** Heatmap displaying the altered BALF metabolites between control and EAE mice (18 dpi) (*n* = 6). **B** Partial least squares discriminant analysis scores plot illustrating the distinct separation of BALF metabolites between the control and EAE groups (R2 = 0.999, Q2 = 0.967) (*n* = 6). **C **Pie chart categorizing the perturbed BALF metabolites. **D** KEGG pathway-based quantitative enrichment analysis highlighting the most significantly affected metabolic pathways in the BALF of EAE mice. Nt: nucleotide

The interplay between microenvironmental metabolites and immune cell behavior suggests that metabolic changes may drive immune adaptations during disease progression [16]. Therefore, we analyzed metabolites present in BALF from control and EAE mice at the peak stage (18 dpi). A total of 140 metabolites were identified, with 28 showing differential abundance (18 upregulated and 10 downregulated in EAE mice) (Fig. 4A). Our analysis revealed significant differences in the BALF metabolome between control and EAE mice (Fig. 4B), characterized by notable alterations across various classes of metabolites, including amino acids (39.00%), lipids (29.00%), nucleotides (11.00%), carbohydrates (7.00%), and cofactors and vitamins (7.00%) (Fig. 4C). Pathway analysis indicated that the most affected pathways involved lipid, amino acid, and nucleotide metabolism (Fig. 4D). Together, these results suggest that EAE induces substantial metabolic remodeling in the lung microenvironment, potentially influencing immune cell function through alterations in lipid, amino acid, and nucleotide metabolic pathways.

### β-oxidation mediates EAE BALF-induced differentiation of effector CD4⁺ T cells

**Fig. 5:**
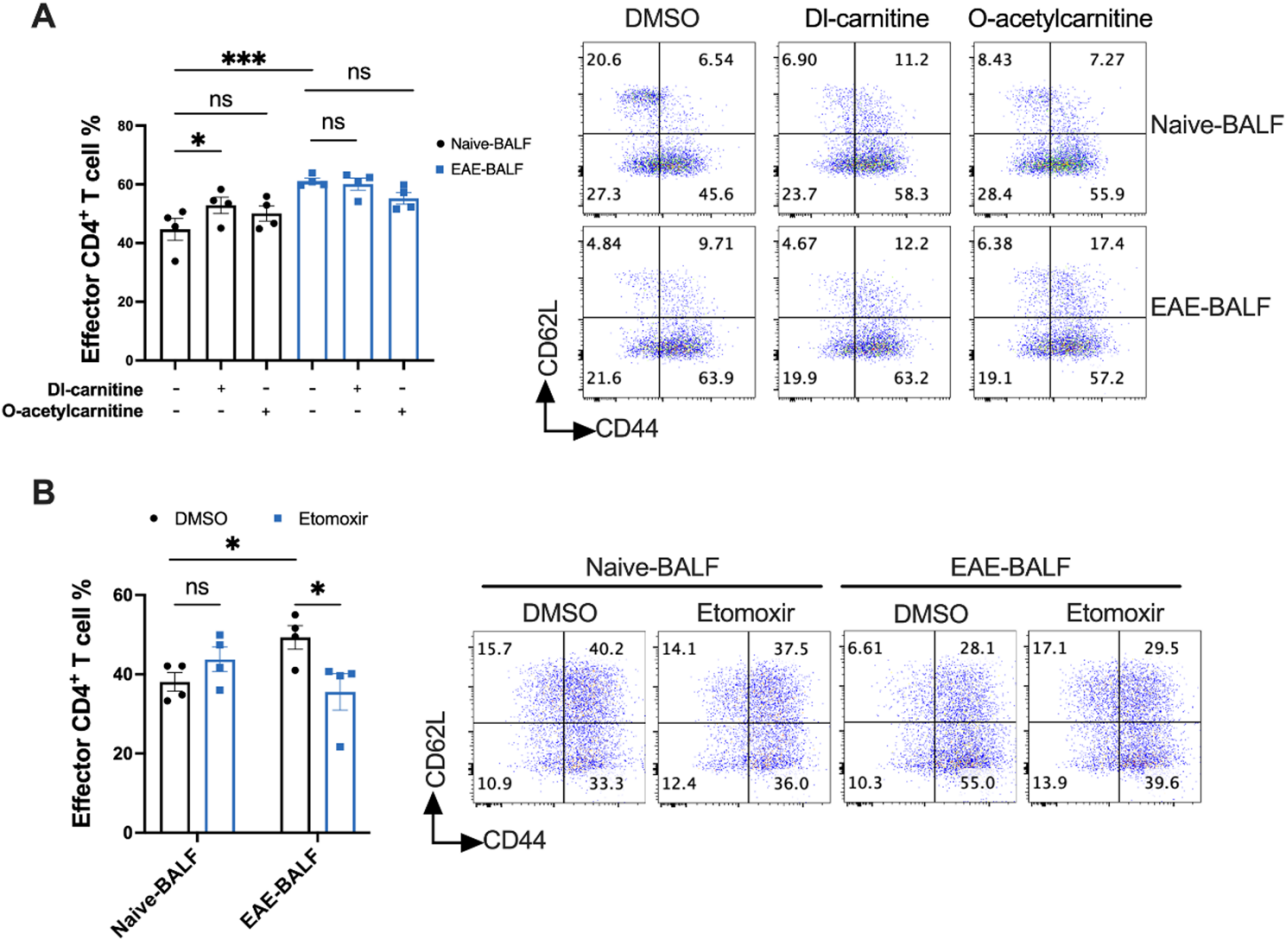
Differentiation of effector CD4⁺ T cells induced by BALF collected from EAE mice is dependent on carnitine-mediated β-oxidation.** A** Naïve CD4⁺ T cells were cultured with BALF from naïve or EAE mice (19 dpi) in the presence or absence of Dl-carnitine or O-acetylcarnitine under anti-CD3e/CD28 stimulation for 24 h. Frequencies and representative flow cytometry plots of effector CD4⁺ T cells are shown (*n* = 4). **B** Naïve CD4⁺ T cells were cultured with BALF from naïve or EAE mice (19 dpi) and treated with or without etomoxir under anti-CD3e/CD28 stimulation for 24 h. Frequencies and representative flow cytometry plots of effector CD4⁺ T cells are shown (*n* = 4). Data are mean ± SEM. Statistical analyses were performed using one-way ANOVA with Tukey’s multiple comparisons test (**A**), and unpaired Student’s t-test (**B**). *P < 0.05 and ***P < 0.001

Given that the lung microenvironment in EAE mice enhances fatty acid uptake and upregulates carnitine transporters in CD4⁺ T cells, we next examined whether specific lipid-related metabolites were altered in the BALF of EAE mice. Notably, BALF from EAE mice contained higher levels of Dl-carnitine and O-acetylcarnitine (Fig. 4A). Dl-carnitine is a mixture of L-carnitine and D-carnitine, while O-acetylcarnitine is an acetylated form of L-carnitine. Both L-carnitine and O-acetylcarnitine are essential for transporting long-chain fatty acids from the cytosol into the mitochondrial matrix for β-oxidation through a process dependent on carnitine palmitoyltransferase 1 [12, 17]. These observations prompted us to investigate whether enhanced carnitine uptake boosts β-oxidation, thereby mediating the differentiation of effector CD4⁺ T cells in the lungs of EAE mice. To test this, naïve CD4⁺ T cells were treated with BALF collected from naïve or EAE mice (19 dpi), in the presence or absence of Dl-carnitine or O-acetylcarnitine. Interestingly, supplementation with Dl-carnitine increased the frequency of effector CD4⁺ T cells in cultures treated with BALF from naïve mice but had no additional effect in the presence of BALF from EAE mice (Fig. 5A), possibly suggesting that the EAE BALF already provides a metabolically carnitine-enriched environment with reduced responsiveness to further supplementation. We further evaluated the contribution of β-oxidation by treating naïve CD4⁺ T cells cultured with BALF from the naïve or EAE mice (19 dpi) with or without the β-oxidation inhibitor, etomoxir. We found that inhibition of β-oxidation significantly reduced the differentiation of effector CD4⁺ T cells cultured with BALF from EAE mice (Fig. 5B), indicating that enhanced β-oxidation is a key mechanism by which the lung microenvironment in EAE mice promotes effector CD4⁺ T cell differentiation.

### The lung metabolome exhibits high similarity to the brain metabolome, but no association with the lung microbiota

**Fig. 6:**
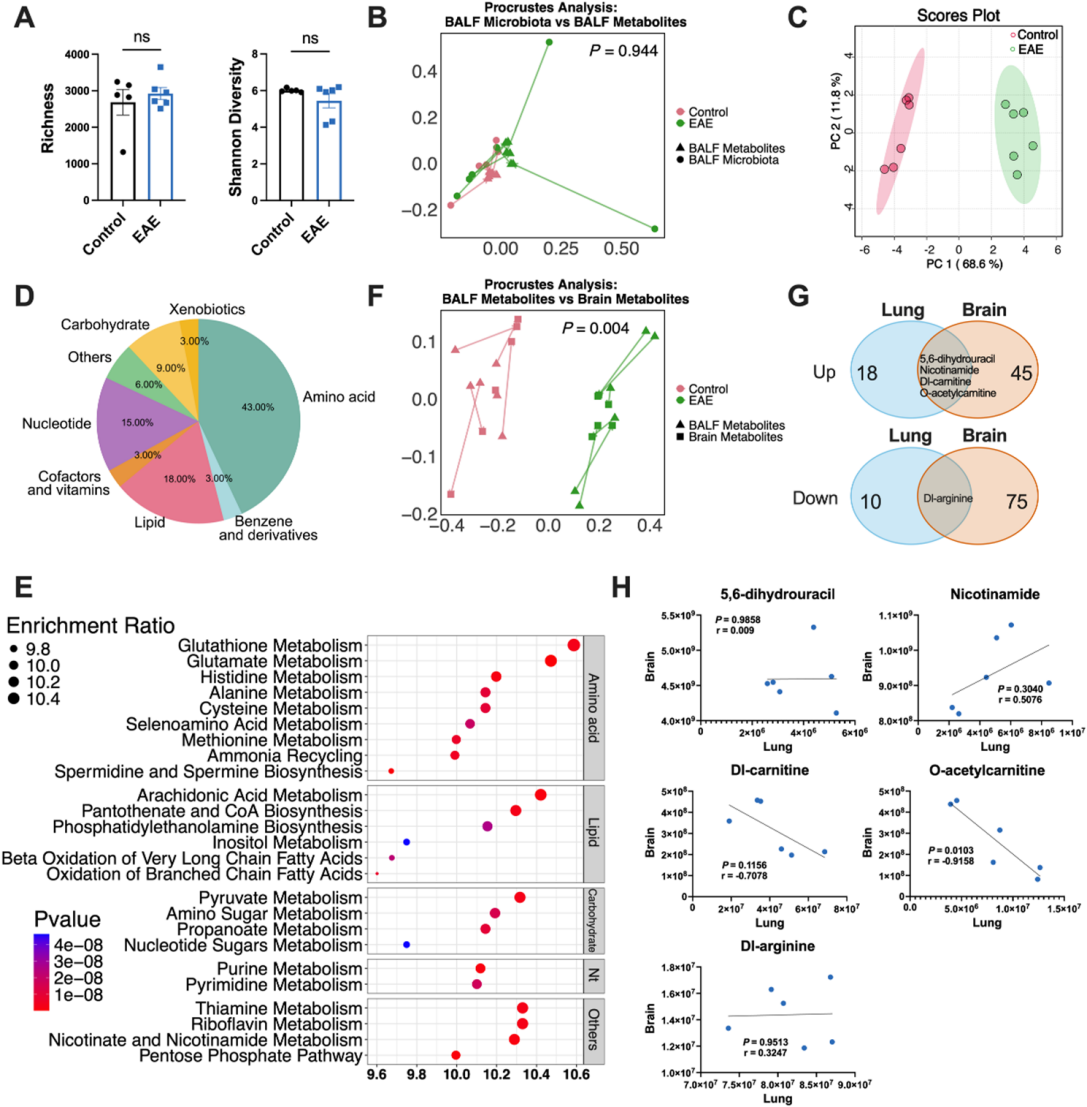
High similarity between lung and brain metabolomes. **A** Richness and Shannon diversity of lung microbiota in control (*n* = 5) and EAE (*n* = 6) mice (18 dpi). **B **Procrustes analysis illustrating similarity between BALF metabolome (*n* = 11) (triangles) and BALF microbiota (*n* = 11) (circles). The length of the edges correlates with the degree of inter-omic data structure similarity within a biological replicate. **C** Partial least squares discriminant analysis scores plot demonstrating the distinct separation of brain metabolites between the control (*n* = 6) (red) and EAE (*n* = 6) groups (green) (R2 = 0.999, Q2 = 0.988). **D** Pie chart categorizing the perturbed brain metabolites. **E** KEGG pathway-based quantitative enrichment analysis illustrating the most significantly affected metabolic pathways in the brains of EAE mice. **F **Procrustes analysis illustrating similarity between BALF metabolome (*n* = 12) (triangles) and brain metabolome (*n* = 12) (squares). **G** Venn diagrams showing the overlap of metabolites that are upregulated or downregulated in the brains and lungs of EAE mice compared with controls. **H **Correlation analysis of selected metabolites in paired brain and lung from individual EAE mice. Data are mean ± SEM. Statistical analyses were performed using unpaired Student’s t-test (**A**), and a permutation-based Procrustes test (**B** and **F**). Statistical significance of the regression coefficients was assessed using t-tests (**H**)

Next, we sought to investigate the sources of metabolic alterations in the lungs of EAE mice. Given the critical role of the microbiome in shaping host metabolic profiles [18–20], we performed metagenomic analysis of BALF samples from the same cohort of control and EAE mice (18 dpi) to determine whether the observed metabolic changes were linked to alterations in the lung microbiota. However, we found no significant changes in lung microbial composition following EAE induction (Fig. 6A), nor any correlation between the lung microbiota and metabolome (Fig. 6B), suggesting that the metabolic reprogramming of the lungs of EAE mice is unlikely to be caused by the changes of microbiota.

Recent studies suggest a lung-brain axis, through which metabolites can mediate communication between these organs [1, 5, 7, 21]. Based on this, we analyzed brain metabolites from the same cohort of mice to explore potential links with lung metabolic changes. Similarly, partial least-squares discriminant analysis identified distinct metabolic profiles in the brains of control versus EAE mice (Fig. 6C). The most significantly affected metabolites were predominantly amino acids (43.00%), followed by lipids (18.00%), nucleotides (15.00%), carbohydrates (9.00%), cofactors and vitamins (3.00%), and xenobiotics (3.00%) (Fig. 6D). Further pathway analysis revealed that the most disrupted pathways in EAE mice were mainly associated with amino acid, lipid, carbohydrate, and nucleotide metabolism (Fig. 6E). Notably, we observed a significant degree of similarity between the BALF metabolome and brain metabolome (Fig. 6F). Importantly, 5,6-dihydrouracil, nicotinamide, Dl-carnitine, and O-acetylcarnitine were upregulated in both the brains and lungs of EAE mice, whereas Dl-arginine was downregulated in both tissues compared with controls (Fig. 6G). To explore potential associations of these metabolites between the lungs and brains in EAE mice, we performed correlation analyses and found that the abundance of O-acetylcarnitine in the lungs was negatively correlated with its levels in the brains, with Dl-carnitine showing a similar negative correlation (Fig. 6H). In contrast, 5,6-dihydrouracil, nicotinamide and Dl-arginine did not exhibit significant correlations between the two organs (Fig. 6H). Overall, these results suggest carnitine-related metabolites may play a role in mediating communication along the lung-brain axis during EAE.

### Inhibition of β-oxidation in the lung reduces EAE severity

**Fig. 7:**
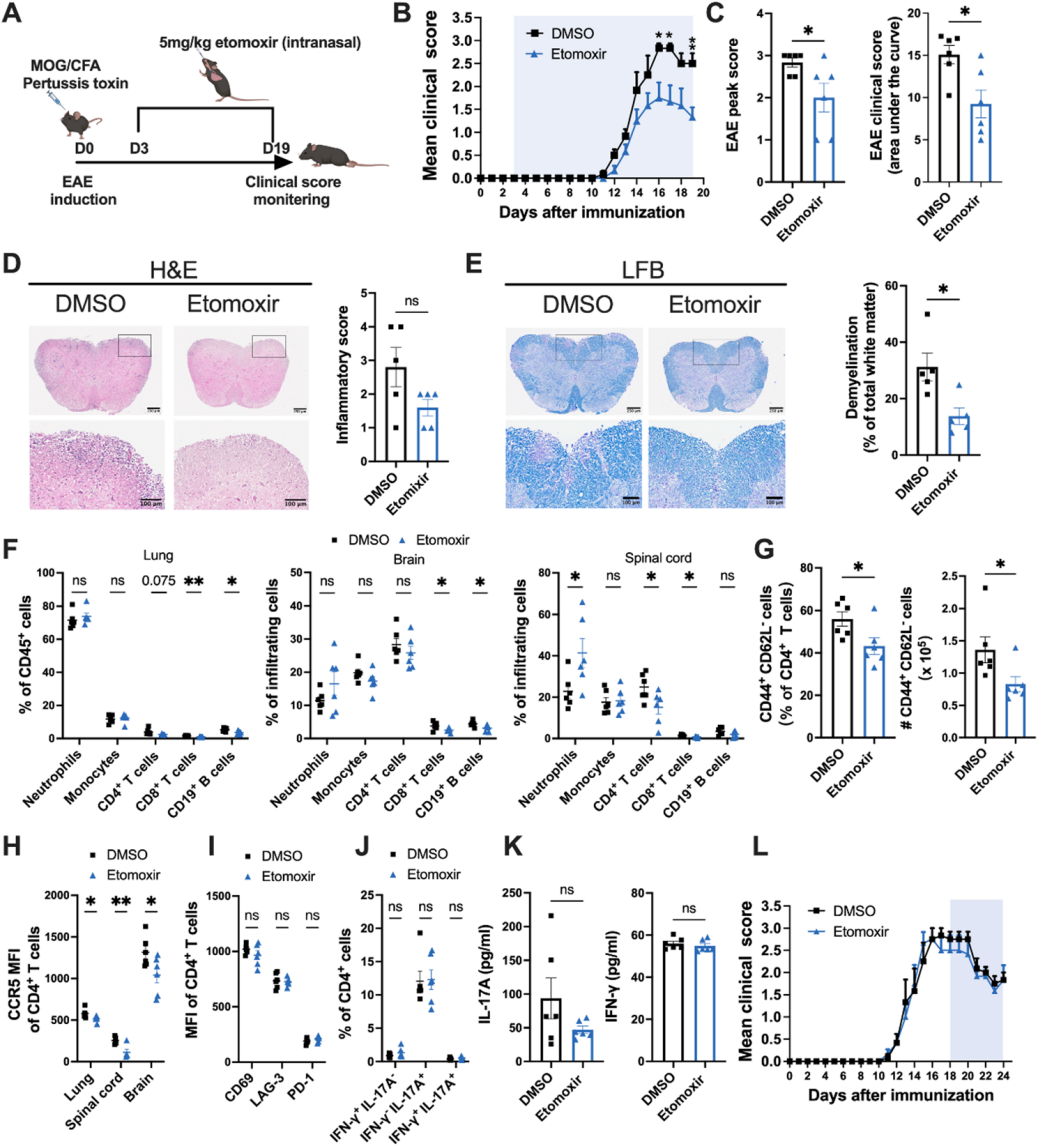
Inhibiting β-oxidation in the lung effectively ameliorates EAE. **A** Schematic illustration of etomoxir treatment and experimental design. **B** EAE clinical scores for MOG_35−55_-immunized mice intranasally treated with 5 mg/kg etomoxir or DMSO starting from day 3 post-immunization (*n* = 6). **C** EAE peak score and quantification of the area under the curve of EAE clinical scores for DMSO- and etomoxir-treated mice (*n* = 6). **D-E** Representative H&E staining images and quantification of the inflammatory score (**D**), and representative LFB staining images and quantification of demyelination (**E**) in the spinal cords from DMSO- and etomoxir-treated groups (*n* = 5). **F **Frequencies of various immune cells in the lungs, brains, and spinal cords of DMSO- and etomoxir-treated EAE mice (*n* = 6). **G** Frequencies and absolute number of effector CD4⁺ T cells in the lungs of DMSO- and etomoxir-treated EAE mice (*n* = 6). **H** MFI of CCR5 in CD4⁺ T cells from the lungs, brains, and spinal cords of DMSO- and etomoxir-treated EAE mice (*n* = 5–6). **I** MFI of activation/exhaustion markers CD69, LAG-3, and PD-1 in CD4⁺ T cells from the lungs of DMSO- and etomoxir-treated EAE mice (*n* = 6). **J **Frequencies of IFN-γ-producing, IL-17A-producing, and IFN-γ- and IL-17A- dual-producing CD4⁺ T cells in the lungs of DMSO- and etomoxir-treated EAE mice (*n* = 6). **K** Serum levels of IL-17A and IFN-γ in DMSO- and etomoxir-treated EAE mice (*n* = 6). **I** EAE clinical scores for MOG_35−55_-immunized mice intranasally treated with etomoxir or DMSO starting from day 18 post-immunization (*n* = 6). Data are mean ± SEM. Statistical analyses were performed using unpaired Student’s t-test (**B**-**J**) and Welch’s t-test (**K**). *P < 0.05, and **P < 0.01

To determine whether pharmacological inhibition of β-oxidation in the lung could modulate EAE progression, we first assessed whether prophylactic treatment with etomoxir could alleviate EAE severity. We intranasally treated the MOG_35−55_-immunized mice with 5 mg/kg etomoxir from day 3 to day 19 post-immunization (Fig. 7A). Remarkably, etomoxir treatment not only delayed disease progression and reduced severity but also mitigated inflammation and demyelination in the spinal cords of EAE mice (Fig. 7B-E). We next assessed how etomoxir treatment affects immune cell composition in the lung and CNS of EAE mice. We found that CD4⁺ T cell frequencies were reduced in the lung and spinal cord, CD8⁺ T cells were decreased in the lung, brain, and spinal cord, and CD19⁺ B cells were reduced in the lung and brain (Fig. 7F). Notably, both the frequency and absolute number of CD44⁺ CD62L⁻ effector CD4⁺ T cells were significantly reduced in the lungs of EAE mice (Fig. 7G). Given the reduced accumulation of CD4⁺ T cells in the CNS, we next examined the expression of CCR5, a chemokine receptor associated with T cell migration into the CNS [22]. CCR5 expression on CD4⁺ T cells was consistently reduced in the lungs, brains, and spinal cords following etomoxir treatment (Fig. 7H), suggesting impaired trafficking potential. We further assessed whether etomoxir affects CD4⁺ T cell activation or differentiation in the lung. No significant changes were observed in the expression of the activation marker CD69 or exhaustion markers (LAG-3 and PD-1) (Fig. 7I). Similarly, these CD4⁺ T cells exhibited similar abilities in producing IFN-γ and IL-17A (Fig. 7J). Consistent with this data, IL-17A and IFN-γ levels remained unchanged in the serum (Fig. 7K). To determine whether lung-targeted metabolic modulation influences disease resolution, etomoxir was administered during the recovery phase of EAE. This treatment did not accelerate disease remission (Fig. 7L), indicating that licensing of CD4⁺ T cells in the lung occurs during the early phase of EAE pathogenesis. Taken together, these results indicate that inhibition of β-oxidation in the lung ameliorates EAE severity and is associated with reduced accumulation of effector CD4⁺ T cells in the lung as well as impaired CD4⁺ T cell trafficking to the CNS.

## Discussion

Effector CD4⁺ T cells are central drivers of pathology in MS and EAE, mediating CNS inflammation and demyelination. Traditionally, these cells are thought to be primed in lymphoid organs such as the spleens and lymph nodes before trafficking to the CNS. However, emerging evidence indicates that CD4⁺ T cells can also be functionally modulated in non-lymphoid peripheral tissues [23, 24]. In particular, the lung has been identified as a potential “licensing” hub, where T cells acquire a migratory phenotype prior to CNS infiltration [9]. Consistent with this concept, we observed a marked expansion of effector CD4⁺ T cells in the lungs of EAE mice. Moreover, our study demonstrates that the lung microenvironment in EAE mice actively promotes effector CD4⁺ T cell differentiation through lipid metabolic reprogramming. This aligns with growing evidence that T cell fate and function are closely linked to cellular metabolic states, with lipid metabolism emerging as a critical regulator of effector differentiation across diverse disease contexts [25–28]. Consistent with this, untargeted metabolomic analyses in patients with MS and EAE models have repeatedly highlighted lipid metabolism as one of the most perturbed pathways in multiple tissues, including plasma, serum, urine, and cerebrospinal fluid [29–33]. Furthermore, studies in lung cancer, breast cancer, and chronic obstructive pulmonary disease all have revealed that alterations in pulmonary lipid metabolism can shape lung immune cell functions and subsequently influence disease outcomes [34–36]. Importantly, our single-cell RNA sequencing analysis further revealed a significant expansion of a *Cxcr6*⁺ effector CD4⁺ T cell subset, which has been previously associated with tissue residency, enhanced migratory capacity, and inflammatory responses, particularly in non-lymphoid tissues [37–39]. Notably, these cells exhibit high expression of the carnitine transporter gene *Slc22a4* (Fig. 2E). Collectively, these findings suggest that lipid metabolism in the lung can influence CD4⁺ T cell responses during CNS autoimmunity.

A major source of lipids within the lung microenvironment is pulmonary surfactant, a lipid-protein complex predominantly composed of phosphatidylcholine (80–85%) and phosphatidylglycerol (10%) [40]. One plausible mechanism underlying the altered lung metabolic profiles in EAE is remodeling of pulmonary surfactant, supported by the pronounced changes we observed in phospholipid biosynthesis-related pathways in BALF from EAE mice. These observations suggest that EAE induction may reshape the pulmonary lipid milieu by altering surfactant composition. Our study further ruled out the possibility that microbiota alterations underlie the observed metabolic changes in the lungs of EAE mice (Fig. 6A, B). This finding points to host-derived mechanisms as the primary contributors to lung lipid remodeling during EAE. A second, non-mutually exclusive mechanism may involve interorgan metabolic communication as previous studies have shown that metabolites derived from other organs can influence lung immune responses and function [41]. For instance, gut microbiota-derived short-chain fatty acids can modulate lung immune responses and metabolic profiles through the gut-lung axis [42] and metabolic dysfunction-associated fatty liver disease is also associated with impaired pulmonary function and chronic lung disease [43]. Given the substantial brain injury that occurs during MS/EAE, we further considered the possibility of lung-brain metabolic crosstalk. Notably, we observed a negative correlation of carnitine-related metabolites, which can be shuttled across different tissues as metabolic intermediates or signaling molecules [44], between the lungs and brains of EAE mice. This finding raises the possibility that brain metabolic alterations may contribute to lung metabolic remodeling during disease. Future studies using longitudinal sampling, metabolic flux analysis, and barrier transport assays will be required to test this hypothesis. Although BALF is a complex biological fluid, our metabolomics, single-cell RNA sequencing data, and functional assays consistently indicate that carnitine-related metabolites are enriched in the lung microenvironment of EAE mice and are likely key contributors to the observed effects. Importantly, carnitine supplementation experiments further support a functional role for this metabolic pathway in promoting effector CD4⁺ T cell differentiation. While other BALF components may also contribute, our data collectively demonstrate that lipid metabolism-related factors are sufficient to recapitulate the observed phenotype.

Several studies suggest that local modulation of the lung can influence CNS diseases, highlighting the lung-brain axis as a potential therapeutic target [5–7, 45, 46]. Our findings further support the lung-brain axis as a therapeutic target in CNS autoimmunity. We demonstrate that inhibition of β-oxidation in the lungs using etomoxir is sufficient to modulate CNS pathology without directly targeting the CNS. While previous studies have shown that genetic [47] or pharmacological [48] inhibition of β-oxidation ameliorated EAE severity, our results highlight the specific contribution of β-oxidation in the lung to disease progression. Although etomoxir can induce *Cpt1a-*independent off-target effects, including oxidative stress at high concentrations [49], oxidative stress has generally been associated with exacerbation of EAE pathogenesis [50], making it less likely to account for the protective effects observed in our study. Nevertheless, we cannot fully exclude potential contributions from such off-target effects. Future studies employing more specific genetic or pharmacological approaches will be important to further delineate the role of fatty acid oxidation in this context. In addition, future studies using single-cell RNA sequencing would provide higher-resolution insights into the cell type-specific effects of etomoxir and help to further dissect the underlying mechanisms. Although our study focused on lipid metabolism, alterations in other metabolic pathways, including amino acid and nucleotide metabolism, in the lungs of EAE mice may also represent potential therapeutic targets [51–54].

## Conclusion

In conclusion, our study uncovers a previously underappreciated role of the lung microenvironment in shaping pathogenic CD4⁺ T cell responses in EAE. Rather than serving as a transit site, the lung provides metabolic cues that guide incoming naïve T cells toward an effector phenotype during EAE development. Importantly, we demonstrate that inhibition of β-oxidation in the lung using etomoxir is sufficient to modulate subsequent CNS pathology, revealing the lung-brain axis as a promising therapeutic target.

## Materials and methods

### Mice and EAE induction

Active EAE was induced in 8-10-week-old C57BL/6 mice of both sexes by subcutaneous injection of 200 µl of 2 mg/ml MOG₃₅-₅₅ peptide (MEVGWYRSPFSRVVHLYRNGK, Thermo Scientific) emulsified in Freund’s complete adjuvant containing 2 mg/ml Mycobacterium tuberculosis H37Ra extract (BD Biosciences, 231141). Mice were intraperitoneally injected with 400 ng of pertussis toxin (Calbiochem, 516560) on days 0 and 2 after immunization. EAE clinical signs were scored daily in a blind manner as previously described [55].

### Single-cell RNA sequencing analysis

CD4⁺ T cells were extracted from our single-cell RNA-sequencing dataset from the lungs and spleens of EAE (19 dpi, score 3) and naïve mice. Dimensionality reduction was performed using principal component analysis (RunPCA) and Uniform Manifold Approximation and Projection (RunUMAP) based on the first 10 principal components. Clustering was conducted with the FindNeighbors and FindClusters functions (resolution = 1.5), resulting in seven transcriptionally distinct subsets defined by key marker genes. Gene Ontology (GO) enrichment analyses were performed using the clusterProfiler package (v4.12.6) based on cluster-specific marker genes. Differentially expressed genes (DEGs) were identified with Seurat’s FindMarkers function using the Wilcoxon rank-sum test and Bonferroni correction. DEGs between CD4⁺ T cell from naïve and EAE mice were filtered using log₂ fold change > 2, adjusted *p*-value < 0.05, and min.pct = 0.1. KEGG pathway enrichment analysis was performed with the clusterProfiler package (v4.12.6). Data visualization was performed using Seurat (v5.3.0), SCP (v0.5.6), scplotter (v0.1.0), and ggplot2 (v3.5.2).

### Sample collection and processing for metabolomic and metagenomic analyses

The whole brains from control and EAE mice were collected on day 18 post-immunization and sent to Beijing Genomics Institute (HKSAR, China) for untargeted metabolomics analysis. The detailed procedure of the metabolic analysis was previously described [56]. Concurrently, BALF was collected from both groups by inserting a catheter into the trachea and flushing the lungs with 3 ml of saline. Half of the BALF was centrifuged at 12,000 × g for 10 min at 4 °C, and the pellet was collected for metagenomic analysis. The remaining half was centrifuged at 1,000 × g for 10 min at 4 °C, and the supernatant was collected for metabolomic analysis.

### Metagenomic data processing

Raw sequence data were quality-filtered using Trimmomatic (v0.39) to remove adapter sequences and low-quality reads (quality score < 30). High-quality reads were further processed with KneadData (v0.12.0) for mouse DNA depletion to reduce host genomic contamination. Taxonomic classification of bacteria was performed on the metagenomic reads using Kraken2 (v2.1.3), an advanced metagenomic taxonomy classifier that employs k-mer-based algorithms. Bracken (v2.5.0) was employed to accurately estimate taxonomic abundances, particularly at the species and genus levels, based on the Kraken2 assignments. Shannon diversity indices were calculated using the R package vegan, and species richness was determined as the number of unique bacterial species in each sample.

### Flow cytometry

Mice were euthanized by lethal anesthesia, and selected tissues (spleen, lung, peripheral blood, brain, and spinal cord) were collected from naïve (untreated) mice and from EAE mice at onset (10 dpi), peak (19 dpi), and recovery (35 dpi) stages, depending on the experimental design. The whole lungs and brains were excised, minced, and incubated in HBSS medium supplemented with 5% FBS (Gibco, A5256801) and 0.04 mg/ml Collagenase Type I (Gibco, 17100017) for 45 min at 37℃. After digestion, tissues were mechanically disrupted, and passed through a 70-µm cell strainer. The spinal cords and spleens were collected and mechanically dissociated through a 70-µm cell strainer. Red blood cells were lysed using ACK lysing buffer (Gibco, A1049201), and the cell pellet was washed and resuspended in the appropriate medium to obtain a single-cell suspension. For surface marker staining, single-cell suspensions were blocked with normal rat serum and stained with fluorescently labeled antibodies against mouse CD45 (Biolegend, 103112), CD11b (BioLegend, 101235), Ly6C (BioLegend, 128033), Ly6G (BioLegend, 127618), CD4 (Biolegend, 100449), CD44 (Biolegend, 103005), CD62L (Biolegend, 104417), CD8 (Biolegend, 100751), CD19 (Biolegend, 115529), CCR5 (Biolegend, 107012), CD69 (Biolegend, 104512), LAG-3 (Biolegend, 125210), and PD-1 (Biolegend, 135214) at 4 °C for 30 min. For intracellular staining, cells were stimulated with Cell Stimulation Cocktail (eBioscience, 00-4970-93) for 5 h, then fixed and permeabilized using the BD Cytofix/Cytoperm kit (BD Biosciences, 554714). Cells were subsequently stained with fluorescently labeled antibodies against mouse IL-17A (eBioscience, 25-7177-82) and IFN-γ (BioLegend, 505850) following the manufacturer’s instructions. Lipid content was measured by flow cytometry after staining cells with 2 µM BODIPY 493/503 (MCE, HY-W090090) at room temperature for 20 min. Fatty acid uptake was assessed by flow cytometry after incubating cells with 2 µM BODIPY FL C16 (Invitrogen, D3821) at room temperature for 20 min. Dead cells were excluded from the analysis using propidium iodide (Sigma-Aldrich, 537060) or Ghost Dye (Tonbo Biosciences, 13–0870). Flow cytometry data were acquired using a BD Celesta flow cytometer (BD Biosciences) and analyzed with FlowJo software (version 10.8.1, BD Biosciences).

### In vitro culture and treatment of naïve CD4⁺ T cells

Naïve CD4⁺ T cells (CD44⁻ CD62L⁺) were isolated from spleens of naïve C57BL/6 mice using the Naïve CD4⁺ T Cell Isolation Kit (Stemcell, 19765). For effector CD4⁺ T cell differentiation, naïve CD4⁺ T cells were stimulated with 5 µg/ml plate-bound anti-CD3e (eBioscience, 16-0031-85) and 2 µg/ml soluble anti-CD28 (eBioscience, 16-0281-82) in the presence of 10% BALF collected from naïve or EAE mice (19 dpi) for 24 h. In carnitine treatment experiments, 1 mM Dl-carnitine (Targetmol, T0743) or 1 mM O-acetylcarnitine (Targetmol, T2563) was added to naïve CD4⁺ T cells treated with BALF collected from naïve or EAE mice (19 dpi) under anti-CD3e/CD28 stimulation for 24 h. For lipid content and fatty acid uptake assays, naïve CD4⁺ T cells were cultured with 10% BALF under anti-CD3e/CD28 stimulation for 5 h or 1 h, respectively. For etomoxir treatment, naïve CD4⁺ T cells were cultured with 10% BALF from naïve or EAE mice (19 dpi) w/o 50 µM etomoxir (MCE, HY-50202 A) under anti-CD3e/CD28 stimulation for 24 h.

### Adoptive transfer of CD4^+^ T cells

Naïve CD4⁺ T cells were isolated from the spleens of naïve C57BL/6 mice and labeled with CFSE. A total 1.5 × 10⁶ CFSE-labeled CD4⁺ T cells were intravenously transferred into either naïve or EAE mice (19 dpi). After 24 h, blood, spleens, and lungs were collected, and the transferred CFSE⁺ CD4⁺ T cells were analyzed.

### In vivo etomoxir treatment

For prophylactic treatment, EAE mice were randomly assigned to two groups: one group received 5 mg/kg etomoxir (MCE, HY-50202 A), while the other group was treated with an equivalent volume of DMSO dissolved in 50 µl PBS. Treatments were administered intranasally beginning on day 3 to day 19 post-immunization. For therapeutic treatment, EAE mice were administered etomoxir intranasally at a dose of 10 mg/kg from day 18 to day 24 post-immunization. Clinical scores were monitored daily.

### Histopathology

Spinal cords were fixed in 4% paraformaldehyde and embedded in paraffin. Serial sections (5 μm) were prepared for hematoxylin and eosin (H&E) staining (Solarbio, G1120) and luxol fast blue (LFB) staining (Abcam, ab150675). After staining, the sections were covers-lipped and scanned with the NanoZoomer S60 Digital slide scanner (Hamamatsu Photonics, C13210-01). Inflammatory scores were calculated as previously described [57]. Demyelination was quantified as the ratio of the demyelinated area to the total white matter area.

### ELISA

Serum levels of IL-17A and IFN-γ in DMSO- and etomoxir-treated EAE mice were quantified using the Mouse IL-17A Precoated ELISA Kit (Dakewe, 1211702) and Mouse IFN-γ Precoated ELISA Kit (Dakewe, 1210002), following the manufacturer’s instructions. Data were collected and analyzed using SpectraMax iD3 (Molecular Devices).

### Statistical analysis

MetaboAnalyst 5.0 was used for multivariate statistical analysis of metabolomics data [58]. Statistical analyses of single-cell RNA sequencing data were performed using R (The R Project for Statistical Computing), with group comparisons conducted using the non-parametric Wilcoxon rank-sum test. The degree of concordance between the metabolome and microbiome data was evaluated by Procrustes analysis and *P* values were calculated by a permutation-based Procrustes test. Other analyses were conducted with GraphPad Prism (GraphPad Software, v10.2.1). Data were first assessed for normality. For normally distributed data, comparisons between two groups with equal variances were performed using unpaired two-tailed Student’s t-tests, while Welch’s t-test was applied for unequal variances. Comparisons involving more than two groups were analyzed using one-way analysis of variance followed by Tukey’s post hoc test. Data are presented as mean ± SEM, and statistical significance was defined as *P* < 0.05. 

## Data Availability

The datasets used and/or analyzed during the current study are available from the corresponding author on reasonable request.
